# Chemical and toxicological assessment of leachates from UV-degraded plastic materials using *in-vitro* bioassays

**DOI:** 10.7717/peerj.15192

**Published:** 2023-04-11

**Authors:** Weike Schwarz, Stina Wegener, Gerhard Schertzinger, Helena Pannekens, Peter Schweyen, Georg Dierkes, Kristina Klein, Thomas A. Ternes, Jörg Oehlmann, Elke Dopp

**Affiliations:** 1 Department of Toxicology, IWW Water Center, Mülheim a.d. Ruhr, NRW, Germany; 2 Center for Water and Environmental Research (ZWU), University of Duisburg-Essen, Essen, NRW, Germany; 3 Federal Institute of Hydrology (BfG), Koblenz, Germany; 4 Department Aquatic Ecotoxicology, Goethe University Frankfurt, Frankfurt am Main, Germany; 5 Medical Faculty, University Duisburg-Essen, Essen, NRW, Germany

**Keywords:** Plastic polymers, Artificial weathering, Plastic additives, Leaching, Endocrine effects, Genotoxicity

## Abstract

The broad use of plastics and the persistence of the material results in plastic residues being found practically everywhere in the environment. If plastics remain in the (aquatic) environment, natural weathering leads to degradation processes and compounds may leach from plastic into the environment. To investigate the impact of degradation process on toxicity of leachates, different types of UV irradiation (UV-C, UV-A/B) were used to simulate weathering processes of different plastic material containing virgin as well as recyclate material and biodegradable polymers. The leached substances were investigated toxicologically using *in-vitro* bioassays. Cytotoxicity was determined by the MTT-assay, genotoxicity by using the p53-CALUX and Umu-assay, and estrogenic effects by the ER*α*-CALUX. Genotoxic as well as estrogenic effects were detected in different samples depending on the material and the irradiation type. In four leachates of 12 plastic species estrogenic effects were detected above the recommended safety level of 0.4 ng 17*β*-estradiol equivalents/L for surface water samples. In the p53-CALUX and in the Umu-assay leachates from three and two, respectively, of 12 plastic species were found to be genotoxic. The results of the chemical analysis show that plastic material releases a variety of known and unknown substances especially under UV radiation, leading to a complex mixture with potentially harmful effects. In order to investigate these aspects further and to be able to give recommendations for the use of additives in plastics, further effect-related investigations are advisable.

## Introduction

There are only rough estimates for the annual global input of plastic into the environment. Scientists estimated that in 2010, 4.8–12.7 million tons of plastic entered the world’s oceans. They predicted an increase by an order of magnitude until 2025 (Jambeck et al., 2015). However, once entered the environment, plastics undergo physical, chemical or biological degradation processes (Andrady, 2011; Gewert, Plassmann & MacLeod, 2015; Gewert et al., 2018; Jahnke et al., 2017). During these processes a wide range of different plastic-associated substances, including monomers, oligomers, additives, as well as degradation and transformation products may be released, contributing to the overall chemical pollution of the respective environment (Teuten et al., 2009; Persson et al., 2013; Hahladakis et al., 2018). The majority of this huge set of substances as well as their effects on the environment and humans is not very well investigated up to now, in particular substances from the last two groups mentioned. On the other hand, toxicological effects of some additives (e.g., phthalates and brominated flame retardants), have been studied more extensively (Oehlmann et al., 2009; Ventura-Camargo & Marin-Morales, 2013; Pivnenko et al., 2016; Pivnenko et al., 2017). Additionally, little is known about effects due to co-occurring substances, so called mixture effects. However, due to the diversity of substances used as additives in plastics, an assessment of the potential associated risks has been difficult to date (Zimmermann et al., 2019; Schaum et al., 2021).

In order to analyze this risk, the “PLASTRAT”-project (http://www.plastrat.de) funded by the German Federal Ministry of Education and Research (BMBF) focused, among other topics, on possible toxicological effects of plastic associated substances. Emphasis was put on the leaching of plastic associated substances after artificial weathering. The effects of leachates from different plastic species (and different weathering scenarios) as well as additives and identified substances from leachates have been investigated and were partly already published by Klein et al. (2021).

Klein et al. (2021) investigated toxicological effects of leachates from twelve artificially weathered plastic polymers using *in-vitro* bioassays (Microtox assay, the AREc32 assay and the yeast-based reporter Assay). The study focused on the impact of UV-radiation on degradation processes, as this weathering scenario was investigated to a lesser extent up to now, especially using *in-vitro* bioassays for toxicity assessment of leachates. The investigated leachates in the study of Klein et al. (2021) showed up to 2896 different substances, many of them were unknown. Furthermore, different effects of the leachates like oxidative stress (85% of investigated substances), baseline toxicity (42%), anti-estrogenicity (40%) and anti-androgenicity (27%) were detected. The number of released plastic-associated substances as well as the effect intensities increased when polymers had been additionally radiated with UV-light. In particular, UV-C radiation led to an increased formation of degradation products due to the higher energy of the short wavelength. These results clearly show that plastic weathering is a relevant scenario, resulting in the release of a complex mixture of plastic-associated substances, posing a potential risk for the environment. The study further highlighted that UV radiation is a relevant weathering scenario that further increases the release or the formation of problematic plastic-associated substances. This may pose a risk not only for the environment itself, but also for humans, for example *via* drinking water produced from bank filtration, groundwater and seawater desalination or *via* sea food and agricultural products. But also food contact materials (oral exposure to leachates), clothes (dermal exposure) and furniture (exposure to released substances *via* inhalation) are of relevance. With regard to food contact materials, however, there is a EU Regulation (Commission Regulation (EU) 2022/1616) that does not allow to use recycled plastic if not demonstrated to be properly decontaminated. To address all the mentioned points and as additional part of the PLASTRAT-Project, the present study focused on estrogenic and genotoxic effects of plastic-associated substances resulting from different artificial weathering scenarios and thereby complements the study of Klein et al. (2021). These two endpoints (estrogenicity and genotoxicity) are highly relevant in the regulation of toxicologically not characterized substances in the German drinking water ordinance under the concept of lifelong tolerable health-related indicator values ((HRIV)/ Gesundheitliche Orientierungswerte, GOW) (Grummt et al., 2013; Grummt et al., 2018).

Thus, we investigated seven petroleum-based plastic species, three recyclates as well as two biodegradable and fully or partly bio-based materials. For each plastic in total four leachates were produced, which underwent artificial weathering, including ‘atmospheric’ and ‘aquatic’ scenarios. Leached chemicals were enriched using solid-phase-extraction and investigated using *in-vitro* bioassays to assess cytotoxicity (MTT assay) as a pre-test for CALUX assays. Estrogenic as well as genotoxic effects (ER*α*-CALUX, p53-CALUX, Umu-assay) were investigated. Moreover, the released plastic-associated substances were analyzed in the leachates by high performance liquid chromatography coupled to high-resolution mass spectrometry (LC-QTOF-MS). Here, signal intensities and number of components were detected and used to compare the different weathering scenarios with respect to the release of plastic-associated substances. Additionally, substances present in the leachates were chemically analyzed using a non-target approach. To the best of our knowledge, this is the first study investigating cyto-/genotoxic and estrogenic effects of substances released from plastic material under weathering conditions.

## Material and methods

### Plastic polymers, artificial weathering and solid phase extraction

Selected plastic polymers , artificial weathering and solid phase extraction (SPE) were identical as published by Klein et al. (2021). Table 1 shows the selected polymer types for this study: polypropylene (PP), polyethylene terephthalate (PET), polystyrene (PS), low-density polyethylene (LDPE) and polyvinylchloride (PVC). In order to also consider supposedly more sustainable products, recyclates (R) of PET, LDPE and PVC, polybutylene succinate (Bio-PBS) as bio-based and a starch blend (SB) as biodegradable plastics were analyzed additionally. The SB is a mixture of petroleum-based (polybutylene adipate terephthalate—PBAT) and bio-based material (thermoplastic starch, glycerin and polylactic acid—PLA) as described in Klein et al. (2021) already. For each plastic category two materials, obtained as pellets with a diameter <5 mm were selected, except for the two PVC products. These PVC samples (transparent plate and sheet pile) were cut into pellet-sized pieces to increase the surface area and thus to achieve similar leaching conditions. The leachable surface area was measured for an aliquot of 100 pellets per sample to the nearest 0.001 mm on each dimension under a stereo microscope (Olympus SZ40) and extrapolated to the tested mass (Klein et al., 2021).

**Table 1:** Selected plastic polymers for the experiments (modified from Klein et al. (2021)).

Type	Sample	Code	Description
1	polypropylene homopolymer	PP-H	pristine pellets
	polypropylene copolymer	PP-C	pristine pellets
2	polyethylene terephthalate amorphous	PET-A	pristine pellets
	polyethylene terephthalate recyclate	PET-R	crystalline, post-consumer pellets
3	polystyrene general purpose	PS-GP	pristine pellets
	polystyrene high impact	PS-HI	pristine pellets
4	low-density polyethylene	LDPE	pristine, crystalline pellets
	low-density polyethylene recyclate	LDPE-R	post-industrial pellets
5	polyvinylchloride amorphous	PVC-A	cuts of transparent plate
	polyvinylchloride recyclate	PVC-R	cuts of sheet pile
6	polybutylene succinate bio-based	Bio-PBS	pristine pellets, biodegradable
	starch blend petroleum-/bio-based	SB	pellets, biodegradable


LDPE-R is a post-industrial recyclate from the packaging industry. It is mechanically recycled plastics and contains additives in contrast to the additive-free LDPE.

Artificial weathering and SPE were performed in duplicates by project partners from Goethe University Frankfurt (Germany). The starch blend (SB) was processed later at IWW in the same way (Wegener, 2020). Pellet number/100 g and surface area (cm^2^/L^−1^) of the decomposed plastic material are given in Table S1. Also, pH and conductivity of the water samples after the treatment processes (T1 = dark control, T2 = UV-C, T3 = UV-A/B, T4 = UVA/B_aq_.) were analyzed and are presented in Table S2.

In short and relevant for the present study: for artificial weathering 100 g of each plastic polymer was treated under four different conditions. The simulated scenarios included ‘atmospheric’ as well as ‘aquatic’ weathering. For ‘atmospheric’ weathering conditions, the test materials were irradiated under laboratory conditions for 24 h by either UV-C at 250 nm wavelength (treatment 2, T2) or UV-A/B at 280–400 nm wavelength (treatment 3, T3). To represent an ‘aquatic’ exposure scenario and to cover volatile compounds eventually lost under T2 and T3, test materials were leached for 24 h in 1.1 L of ultrapure water during UV-A/B irradiation at 280–400 nm (T4). A corresponding dark control (T1) was treated as T4 without irradiation. Further weathering conditions e.g., temperature, UV-intensities, lamp characterization and calculation of the simulated irradiance time during weathering are described in Klein et al. (2021).

SPE was performed with 1 L leachates using Telos C18/ENV cartridges (Kinesis GmbH, Wertheim, Germany) to retain hydrophobic compounds (Abbas et al., 2019). Loaded and dried cartridges from one duplicate were sent to IWW (Mülheim, Germany). After elution using acetone and methanol (both, VWR International GmbH, Hannover, Germany), the extracts were resolved in dimethyl sulfoxide (DMSO, VWR International GmbH, Hannover, Germany) to an enrichment factor of 5,000. Extracts were stored at −20 ° C until testing. Previously, 200 µL of the corresponding eluate were stored at −20 °C for chemical analysis at the Federal Institute of Hydrology (Koblenz, Germany).

### Cell culture

p53- and ER*α*-transfected human osteosarcoma cell line U2-OS (BioDetection Systems BV, Amsterdam, Netherlands) were cultured in Dulbecco’s modified eagle medium with nutrient mixture F-12 (DMEM/F12; Thermo Fisher Scientific, Waltham, MA, USA) and phenol red (c.c.pro GmbH, Oberdorla, Germany). The medium was supplemented with 8.0% fetal calf serum (FCS), 0.6% Gentamicin and 1.0% non-essential amino acids (MEM-NEAA, all from c.c.pro GmbH). Both cell types were cultured at conditions of 37 °C, 97% humidity and 5% CO_2_ atmosphere (CO_2_-Incubator INC 153, Memmert, Nussloch, Germany).

For CALUX-assays, cells were seeded in DMEM low glucose (Sigma-Aldrich, Germany) medium (free of phenol red supplemented with 4.8% stripped FCS (BDS), 1.6% gentamicin (c.c.pro), and 1.0% MEM-NEAA (c.c.pro), at a density of 1 × 10^4^ cells in 100 µL medium per well of 96-well-plates. ER*α*-cells were incubated under the above conditions for 24 h and p53-cells for 48 h to allow adherence and confluent growth before exposure to the respective sample.

### Cytotoxicity

The MTT assay is used to determine the metabolic activity of eukaryotic cells and is based on the reduction of the soluble, yellow tetrazolium salt 3-(4,5-dimethylthiazol-2-yl)-2,5-diphenyltetrazolium bromide (MTT) to a blue-purple, insoluble formazan by dehydrogenases localized in the mitochondria of the cells. After cell lysis, formazan can be quantified photometrically. The amount of formazan is proportional to the number of metabolically active eukaryotic cells.

In detail, cells were exposed to different concentrations of the samples for 24 h. 10% DMSO served as a positive control. Subsequently, cells were washed and stained with MTT-solution (5 mg MTT/ml in PBS, Merck KGaA, Darmstadt, Germany). After 2 h of incubation, cell lysis (lysis solution: 99.4 ml DMSO, 0.6 ml acetic acid, 10 g sodium dodecyl sulphate, Merck KGaA) was performed and absorbance at 595 nm was measured using the Spark 10M plate reader (Tecan Deutschland GmbH, Crailsheim, Germany).

The MTT-assay was applied as pre-test in order to avoid false negative results in the CALUX-assays using eukaryotic cell lines. Only the concentration of the respective polymer extract or substance classified as non-cytotoxic with the MTT assay (cell viability >70% according to ISO standard (ISO 10993-5, 2009)) was applied in the CALUX assays to determine estrogenic (ER*α*) and genotoxic (p53) activity (Mosmann, 1983).

### Estrogenicity

The ER*α*-CALUX-assay, used for the detection of estrogenic effects was performed according to ISO 19040-3 (2018). The assay is used to detect receptor-mediated estrogenic activities. “CALUX” stands for chemical activated luciferase gene expression. The human osteosarcoma cell line U2-OS (BioDetection Systems BV, Amsterdam, NL) is genetically modified. The reporter gene luciferase has been coupled to the estrogen receptor gene (ER*α*). In case an estrogen-active substance binds to the ER*α* receptor in the cell, the corresponding gene is read and consequently also the reporter gene. Thereupon luciferase is produced, which reduces the added luciferin to oxyluciferin under the emission of light. The luminescence intensity correlates directly with the amount of substance bound to the receptor. To determine estrogenicity equivalent concentrations, a 17*β*-estradiol (E2) standard series (0–91 ng E2 equivalents per litre, EEQ/L) is included in each assay, which allows the expression of results as ng EEQ/L (BioDetection Systems, 2014; Besselink, 2015).

24 h after seeding in assay medium, adherend and confluent cells were exposed to the respective samples for another 24 h. This was followed by 15 min cell lysis (lysis-buffer, 500 ml cell culture water, 3 g Tris base, 2 mL DTT, 0.73 g CDTA, 100 mL Glycerol, 10 mL Triton X-100; Sigma-Aldrich) and subsequent luciferin (Glow Mix, BDS) addition. Immediately afterwards, luminescence was measured using a Spark 10M plate reader.

A statistical analysis using One-way ANOVA und Tukey’s *post-hoc* test (Prism; GraphPad, San Diego, CA, USA) was performed in order to proof if estrogenic effects measured in the plastic leachates were statistically different from the process control.

### Genotoxicity

#### p53-CALUX

The p53-CALUX-assay was performed to determine the genotoxic potential in eukaryotic cells and follows the same principle as explained for the ER*α*-CALUX-assay. Here, the U2-OS cell line has been genetically modified in such a way that the reporter gene luciferase has been coupled to the tumour suppressor gene p53. The latter is transcripted into proteins after DNA damage and thereby responsible for cell cycle control by regulating other genes involved in apoptosis and DNA repair. If the DNA in a cell is damaged, the translation of p53 starts followed by luciferase production. The luminescence intensity resulting from the reduction of added luciferin is used to indirectly determine the relative induction factor of p53.

Forty-eight hours after seeding in assays medium, adherend and confluent cells were exposed with and without the rat liver extract S9 (liver extract Arochlor in KCL containing 2.25 µL Glucose-6-phosphate dehydrogenase, and 300 µL of 20 mM NADPH, 300 mM glucose-6-phosphate, 500 mM magnesium chloride and S9 in 1.8 mL medium, Trinova Biochem GmbH, Giessen, Germany) to account for possible metabolization of the sample ingredients. In the approach without liver extract, cells were exposed for 24 h. For the genotoxicity detection after metabolic activation 20 µL of the S9 mix were added. After 3 h of incubation exposure media containing S9 mix and sample was replaced by assay medium and cells were incubated for another 21 h. Cyclophosphamide (CPA) served as positive control for the approach with S9; actinomycin D (ACTD) was used in the approach without S9 (both Merck KGaA). After an incubation time of 24 h cell lysis, luciferin addition and luminescence measurement were performed analogue to the ER*α*-CALUX-assay.

The fold-induction per well was calculated by dividing the average Relative Light Units (RLU) level of the tested compound by the average RLU of the solvent control DMSO. Actinomycin-D was used as a positive control and tested compounds were considered “positive” when the response of at least one concentration was above the determined 1.5-fold induction threshold and SD <20% (Van der Linden et al., 2014).

Finally, the IF (induction factor) relative to the negative control was calculated. Plastic leachates were judged to be genotoxic if the IF was greater than or equal to 1.5 in at least two of three test replicates and on average.

#### Umu-assay

The Umu-assay was performed with the genetically modified bacterium *Salmonella typhimurium* TA1535/pSK1002 (Moltox, Boone, USA) following DIN standard (DIN 38415-3, 1996). Genotoxins activate the so-called UmuC gene, which belongs to the SOS repair system of the cell and counteracts damage to the bacterial genetic material. Genetic modification was used to couple the umuC gene with the lacZ gene, which is responsible for the formation of *β*-galactosidase. This enzyme converts the colorless o-nitrophenyl-*β*-D-galactopyranoside (ONPG, Merck KGaA) to the yellow o-nitrophenol which allows the genotoxic potential of a sample to be determined indirectly by a color reaction at 420 nm. The result is expressed as the induction rate (IR) of the enzyme, which corresponds to the increase in absorbance at 420 nm relative to the negative control. The IR is dependent on the growth rate previously determined at 600 nm. If the growth rate of a sample is less than 0.5 compared to the negative control, the results are disregarded due to cytotoxicity.

An exponential growth of the bacterial culture overnight was measured by optical density measurements at 600 nm (Spark 10M plate reader). Bacteria were exposed to the samples for 2 h. Final concentrations of the different extracts tested were 0.67, 1.34, 2.67 and 5.34 g P-EQ /L (SB 0.37 to 2.94 g/L P-EQ). P-EQs are plastic-equivalents based on the amount of plastics used during the leaching experiment in a defined water volume. Exposure of bacteria was performed both with and without the rat liver extract S9 (liver extract Arochlor in KCL) to account for genotoxic effects following metabolization of sample ingredients. As a positive control, 4-nitroquinoline N-oxide (4-NQO) was used in the test batch without S9 and 2-aminoanthracene (2-AA) in the batch with S9 (both Xenometrix, Allschwil, Swiss). To determine the growth rate during exposure, 1:10 dilution was used and optical density was measured immediately after 2 h. For detection of genotoxic effects, 30 µL of the treated bacterial solution were suspended in pre-warmed 120 µL B-buffer (containing 10.1 g disodium hydrogen phosphate dihydrate, 2.75 g sodium dihydrogen phosphate hydrate, 0.38 g potassium chloride, 0.13 g magnesium sulphate and 0.5 g SDS per 500 mL distilled water; and freshly added 0.27% *β*-Mercaptoethanol; Sigma, Munich, Germany) and 30 µL ONPG (o-Nitrophenyl-*β*-D-galactopyranoside; Sigma, Munich, Germany) solution (22.5 mg ONPG in 5 mL Phosphate buffer) for 30 min at 28^∘^C. Than 120 µL stop reaction solution, containing 53 g disodium hydrogen carbonate per 500 mL distilled water (Sigma, Munich, Germany) was added and the extinction was determined photometrical at 420 nm (Spark 10M plate reader). Finally, the induction ratio (IR) relative to the negative control was calculated. Depended on the sample volume in total only two replicates were performed. Calculations of the IR were carried out according to the International Standard ISO 13829 (ISO 13829, 2000) and DIN 38415-3 (DIN 38415-3, 1996). According to the ISO and DIN Standards, plastic leachates were judged to be genotoxic if the IR was greater than or equal to 1.5 in both of the two test replicates.

### Chemical analysis

**Figure 1:**
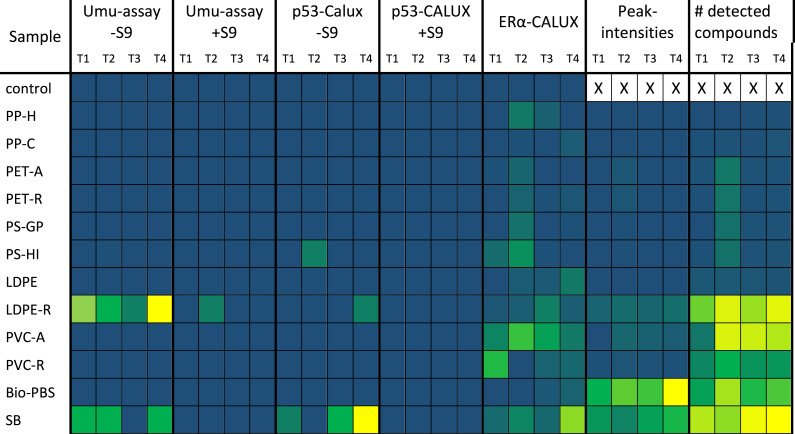
Relative toxicological activities of plastic leachates in all performed bioassays shown as heat map. T1, dark control; T2, UV-C; T3, UV-A/B; T4, UV-A/B_aq._; yellow, 100% and equivalent to highest measured effect; dark blue, 0% and equivalent to lowest measured effect (green in between).

The eluates of Solid Phase Extraxtion (SPE) were measured *via* HPLC-HR-MS as described previously in detail by Klein et al. (2021). Analysis was performed using a TripleTOF 6600 (SCIEX) coupled with an ESI source to a binary HPLC instrument (1260 Infinity, Agilent) equipped with a reversed phase C18 column (Zorbax Eclipse Plus, 2.1 mm ×150 mm, 3.5 µm, Agilent). As eluent a water-acetonitril gradient was used, acidified with 0.1vol% formic acid at a flow of 300 µL min^−1^. The method used both ESI(+) and ESI(-) ionization mode (scan mode 100–1200 Da). Samples were diluted 1:1 with ultrapure water and injection volume was 50 µL (Klein et al., 2021). The data analysis was processed according to a non-target approach as described by Jewell et al. (2020) and Köppe et al. (2020). After data acquisition, peak picking, componentization, alignment of the components and blank correction, the components and their intensities were summed for each sample and ionization mode. The data are summarized in Table S3 of Supplementary Information. The total count and intensity of all detected components were normalized according to the highest and lowest values and displayed in the heat map (see Fig. 1).

## Results

### Cytotoxicity

In order to determine cytotoxic effects of the different plastic leachates on eukaryotic cells and to determine the non-cytotoxic concentrations to perform the CALUX assays on estrogenicity and genotoxicity, the MTT assay was performed using ER*α*- and p53-cells. Test concentrations in the CALUX-assays were chosen based on the results of the MTT-assay.

Two different leachate concentrations were tested in the MTT assay with ER*α*-cells: 178 and 445 g/L P-EQ (starch blend pellets (SB) 98 and 196 g/L P-EQ). At the highest concentration tested, cytotoxic effects (<70% cell viability) were measurable in some plastic leachates (see Fig. S1). At a concentration of 178 g/L P-EQ (SB: 196 g/L P-EQ) leachates from all treatments (T1–T4) did not show cytotoxic effects. Therefore, the ER*α*-CALUX was performed with leachate concentrations of 178 g/L P-EQ (SB: 196 g/L P-EQ), in order to be able to compare the results of the different plastic polymers as well as the different treatments.

In the MTT-assay with p53-cells, three different concentrations of the leachates were tested: 445, 890 and 1780 g/L P-EQ (SB: 392, 979 and 1958 g/L P-EQ). The highest two concentrations showed cytotoxic effects in most of the leachates (see Fig. S2). At the lowest tested concentration leachates from all treatments (T1–T4) did not show cytotoxic effects. Therefore, the p53-CALUX was performed with leachate concentrations of 445 g/L P-EQ (SB 1958 g/L P-EQ) in order to be able to compare the results of the different plastic polymers as well as the different treatments. Using the same P-EQ concentrations allowed a ranking of the investigated polymers with respect to the potential to release estrogenic and genotoxic compounds as a consequence of weathering.

### Estrogenicity

The ER*α*-CALUX was performed with exposure concentrations of the plastic leachates of 178 g/L P-EQ (196 g/L P-EQ for SB). As the procedural blanks (pb) of the SPE using Telos C18/ENV cartridges (Kinesis GmbH) showed slight estrogenic effects (0.29 ng EEQ/L), this background signal was subtracted from results of the leachates, except for SB (no background signal was found here).

The results of the different treatments are shown in Table 2. All values in bold exceed the recommended limit value of 0.4 ng EEQ/L for surface waters (Kienle et al., 2015). Compared to the procedural blank significantly different estrogenic effects of 0.63, 0.58 and 0.94 ng EEQ/L were detected for the PVC-A leachate after UV-C treatment (T2), the PVC-R leachate of the dark control (T1) and the SB leachate after aqueous UV-A/B exposure, respectively. For PVC-A leachate after UV-A/B treatment (T3), an estrogenicity level of 0.44 ng EEQ/L was detected. However, the difference to the procedural blank was not statistically significant. In general, EEQ concentrations were higher in leachates of plastic polymers treated with UV-radiation (except for PVC-R). For SB, estrogenic effects were solely found after the “aquatic” weathering scenario (T4). Estrogenic effects of the different leachate samples at different treatment procedures (T1–T4) are shown in Figure S3.

**Table 2:** 17*β*-Estradiol equivalent concentrations (ng EEQ/L) of plastic leachates (see Table 1) after T1–T4 treatments measured with the ER*α*-CALUX (exposure time 24 h, mean of three replicates after subtracting the background signal, pb: procedural blanks).

**Leachates**	**Estrogenicity, ng EEQ/L**			
	**T1**	**T2**	**T3**	**T4**
PP-H	<pb	0.23	0.10	<pb
PP-C	<pb	0.02	<pb	0.09
PET-A	0.02	0.12	0.01	<pb
PET-R	0.01	0.11	0.02	0.04
PS-GP	<pb	0.19	0.01	0.02
PS-HI	0.16	0.35	0.02	<pb
LDPE	<pb	0.07	0.06	0.23
LDPE-R	0.04	0.08	0.26	0.08
PVC-A	0.29	0.63^**^	0.44^*^	0.23
PVC-R	0.58^***^	<pb	0.11	0.14
Bio-PBS	<pb	<pb	0.05	0.11
SB	0.29	<pb	<pb	0.94^***^

**Notes.**

* < 0.05.

** significant (0.01 > *p* > 0.001).

*** highly significant (*p* < 0.001) compared to the procedural blank tested with one-way ANOVA and Tukey’s *post-hoc* test.

SD < 25%.

### Genotoxicity

The p53-CALUX was performed with exposure concentrations of the plastic leachates of 445 g/L P-EQ (1958 g/L P-EQ for SB). Plastic leachates were judged to be genotoxic if the induction factor (IF) was greater than or equal than 1.5 in at least two of three test replicates and on average (Van der Linden et al., 2014). A genotoxic potential could be determined in leachates of three plastic species and solely in assays performed without metabolization of substances (Table 3). PS-HI was genotoxic after UV-C treatment (T2, IF = 1.55), LDPE-R after “aquatic” weathering with UV-A/B (T4, IF = 1.52) and SB after all weathering scenarios, except UV-C treatment (T1, IF = 1.50; T3, IF = 1.61; T4, IF = 1.89). Hence for SB the threefold concentration (1958 g/L P-EQ) compared to other leachates was tested. The remaining leachates were not genotoxic as the mean IF was either <1.5 or 2 of 3 replicates were negative. The comparison of test results with and without S9 (metabolic activation) is shown in Figure S4. Significant effects were always detectable without S9 supplementation.

**Table 3:** Genotoxic effects of leachates in the p53-CALUX without addition of S9. Plastic leachates were judged to be genotoxic if the induction factor (IF) was greater than or equal to 1.5 in at least two of three test replicates and on average. A minus (−) indicates an IF < 1.5.

Leachates	Genotoxic potential (IF ≥ 1.5)			
	T1	T2	T3	T4
PP-H	–	–	–	–
PP-C	–	–	–	–
PET-A	–	–	–	–
PET-R	–	–	–	–
PS-GP	–	–	–	
PS-HI	–	1.55	–	–
LDPE	–	–	–	–
LDPE-R	–	–	–	1.52
PVC-A	–	–	–	–
PVC-R	–	–	–	–
Bio-PBS	–	–	–	–
SB	1.50	–	1.61	1.89

As second genotoxicity assay, the Umu-test was applied. Here, plastic leachates were tested in 4 concentrations ranging from 0.67 to 5.34 g P-EQ/mL. (SB 0.37 to 2.94 g/mL P-EQ). Samples were judged to be clearly genotoxic if the induction rate was greater than 1.5 for both test replicates. The results, indicating the lowest genotoxic concentration with or without S9, are presented in Table 4. All obtained results with and without S9 can be found in Newe (2019) and Wegener (2020) and in Figure S5.

**Table 4:** Genotoxic effects of leachates in the Umu-test indicating the lowest concentration still genotoxic with (+S9) and without (−S9) addition of S9. A minus (−) indicates an IR < 1.5 in both replicates. A plus (+) indicates an IR ≥ 1.5 in one of the two replicates.

Leachates	Genotoxic potential (IR ≥ 1.5)			
	T1	T2	T3	T4
PP-H	−	−	−	−
PP-C	−	−	+	−
PET-A	+	–	+	−
PET-R	−	+	+	+
PS-GP	+	−	−	+
PS-HI	+	−	−	−
LDPE	−	−	−	−
LDPE-R	1.30 g/ml, −S9	2.67 g/ml, −S9 5.34 g/ml, +S9	5.34 g/ml, −S9	0.67 g/ml, −S9
PVC-A	+	+	+	+
PVC-R	−	+	+	−
Bio-PBS	+	+	+	−
SB	2.94 g/ml, −S9	2.94 g/ml, −S9	+	2.94 g/ml, −S9

The LDPE-R leachates were genotoxic after every type of weathering scenario and after “aquatic” weathering with UV-A/B (T4) already at the lowest tested concentration of 0.67 g/mL P-EQ. The leachates of the weathered SB also exhibited genotoxic potential after T1, T2, and T4. A genotoxic effect after metabolization of substances present in the leachates occurred only for LDPE-R in the dark control (T2).

### Comparison of the toxicological potential of different plastic species after weathering

For comparison of the biological activity of the different investigated plastic leachates, a heat map was created based on the bioassay results of this study (Fig. 1). The heat map shows clearly that leachates of LDPE-R have the highest genotoxic potential (Umu-assay) after T4 treatment. PVC-A and PVC-R show the highest estrogenic effects at T1 and T2 treatment, respectively. SB is genotoxic as shown in the p53-CALUX without S9-Mix (T1: IF = 1.50; T3: IF = 1.61; T4: IF = 1.89) as well as in the Umu-assay without S9 (T1, T3, T4). Leachates of PS-HI were also genotoxic in p53-CALUX without S9 (T2 UV-C, IF = 1.55) (see Fig. 1 and Fig. S4/S5).

### Chemical analysis

As already discussed in Klein et al. (2021), LC-QTOF results can be used to get an overview about the complexity of the compounds present in the leachates and for a rough estimation of the released amounts. Informative value of intensities and comparability of different plastic samples is very limited, due to high differences in detector response for different molecules in LC-MS analysis. However, the data revealed obvious differences in leachates from the plastic samples and UV induced changes in the leaching behavior. Fig. 1 illustrates counts and intensities of components found in the plastic eluates.

LDPE-R, both biodegradable plastics and both PVC samples released the highest number of compounds (between 1012 (PVC-A, T1) and 4873 (SB, T4), see Tab. S3). PP-H released the fewest number of compounds (between 37 (T2) and 78 (T4); Tab. S3). A clear increase of counts caused by UV irradiation could be observed for LDPE-R and PVC-A. For Bio-PBS and SB only a small effect and for the other plastics no effect could be observed.

## Discussion

This study aimed to investigate if chemicals present in plastics are increasingly released after UV-weathering and if the released chemicals can be interlinked with the observed toxicological effects. Therefore, 12 plastic species of PP, PET, PS, LDPE, PVC, Bio-PBS and SB materials were artificially weathered by UV-C, UV-A/B, UV-A/B irradiation in water and a dark control. Our results demonstrate that leachates of plastic materials can exhibit detectable cytotoxic and genotoxic effects as well as endocrine activity.

The number of component counts determined under the simulated weathering conditions increased for most of the samples. Only the SB and LDPE-R samples leached fewer chemicals after each UV treatment or solely after ‘atmospheric’ weathering, respectively. As already pointed out by Klein et al. (2021), most of these released chemicals seemed to be formed due to degradation during the simulated weathering as they represent low-molecular fragments. Accordingly, both the number and the amount of the components generally increase after the UV treatments.

Light stabilizers and other additives were not separately measured. Also, it should be considered when analysing the study results that plastic materials can be produced following different manufacturing processes, using different conditions and formulations. This may result in differences in the leachate profiles and in its toxicity following UV irradiation or environmental degradation.

It was beyond the scope of our study to identify the main drivers for the observed toxicities because this would require an effect-directed analysis. We have not primarily focused on identifying chemicals, but future studies should consider this to identify unknown substances that are responsible for adverse effects. A specific identification of the plastic-associated chemicals also went beyond the scope of our study. However, we were able to identify several components (see Klein et al. (2021)), including stabilizers, plasticizers, organophosphorus compounds, antioxidants and benzoic acids. Many compounds were detected after several treatments. We have also identified many degradation products, particularly for the biodegradable plastics, but also in these cases most of the detected compounds remained unknown.

As part of the BMBF-funded PLASTRAT project (http://www.plastrat.de) our study complements the investigations of Klein et al. (2021). Klein et al. (2021) showed in their study that low-density polyethylene recyclate (LDPE-R), starch blend (SB), bio-based polybutylene succinate (Bio-PBS) and polyvinyl chloride (PVC) triggered toxicological endpoints measured with the Microtox assay, the AREc32 assay and the yeast-based reporter assay, whereas polyethylene terephthalate (PET), polystyrene (PS), polypropylene (PP) and LDPE caused little or no effects. These results fit to our investigations, where LDPE-R and SB caused the strongest effects measured with the MTT-assay, the ER*α*-CALUX, the p53-CALUX and the Umu-assay.

Regarding bioassay testing the German Federal Environment Agency (UBA) recommends a biotest battery when evaluating genotoxic substances in drinking water (Grummt et al., 2018). Following the chemical identification of the compound (if possible), a combination of genotoxicity tests based on bacteria and human cells is recommended—the UBA suggests the micronucleus assay, the Ames test and the UmuC test (Grummt et al., 2018). In our study a combination of the bacterial UmuC test and the human U2-OS cell based p53-CALUX was chosen. Both tests examine the potential of a substance to cause DNA-damage. The application of *in-vitro* bioassays in quality assessment of water samples is an established procedure by now (e.g., Dopp et al., 2019; Dopp et al., 2021; Pannekens et al., 2019; Jia et al., 2019; Gehrmann et al., 2016; Escher et al., 2014).

One interesting observation in our study was, that all investigated leachates did not show genotoxic effects in p53-CALUX when S9-mix was added. Without S9-mix leachates of PS-HI, LDPE-R and SB showed positive results. A possible explanation could be a metabolic transformation and detoxification of the substances in the leachates by the liver extract (S9-mix). The metabolic transformation of substances by liver enzymes is described in Grant (1991). A similar study was conducted by Souza et al. (2020). These authors used the comet assay and the micronucleus test for detection of genotoxic effects in human hepatocarcinoma cells (HEP-G2) caused by aqueous extracts of soil samples containing PBAT mulch films (as used in agriculture) with and without additives. The material of the PBAT films is comparable to the starch blend (SB) pellets tested in this study. The results of Souza et al. (2020) were also negative in both test systems.

The ER*α*-CALUX assay performed in this study is based on the binding of substances to an estrogen receptor. In addition to receptor-dependent processes, there are also receptor-independent processes that can affect endocrine functions. These include, for example, the inhibition of enzymes that are responsible for the production, conversion or elimination of steroid hormones. As a receptor-independent test, the steroidgenesis assay, which determines the effect on the production of 17*β*-estradiol and testosterone, can be performed additionally (Hecker & Giesy, 2008). The GOW concept of the German Federal Environment Agency recommends the use of a steroidgenesis assay in addition to receptor-mediated tests (Grummt et al., 2018). In further toxicological investigations of leachates from plastic material the H295R Steroidogenesis assay should be applied to cover also receptor-independent effects.

The chemical analysis revealed a release of up to 4873 substances from plastic material, which shows the complexity of the subject. Identification of single compounds and linkage to toxicological effects are extremely difficult and time-consuming (Schertzinger, 2020). This task is out of scope of this study and should be done in further studies in order to determine yet unknown substances responsible for adverse effects (Klein et al., 2021). However, in some cases such as PVC-A or LDPE-R increased counts and intensities go along with increased toxicological effects. Beside detected cytotoxic effects, some of the leached substances expressed also genotoxic and/or endocrine disrupting activity. The combination of these effects (mixture toxicity) makes a risk assessment quite complicated. The US EPA suggests a weight-of-evidence hazard index (HI) based approach to predict joint toxicity of chemical mixtures (Kumari & Kumar, 2020). This would be an issue for further research.

The solid phase extraction with a silica-based sorbent (C18) was used in this study for treatment of the water samples because it was considered as a suitable method to extract estrogen-like compounds from the water phase. However, the use of this type of extraction in relation to water in contact with plastics has been the subject of controversial discussion in recent years. According to Bach et al. (2012) improper extraction or sample treatment could lead to false-positive or false-negative estrogenic effects in bioassays. An alternative to solid-phase extraction is the liquid-liquid extraction, which can be considered when testing leachates. Moreover, the tested leachates contain mixtures of substances as described above already. Anti-estrogenic substances can mask estrogenic effects and can lead to false negative results (ISO 19040-3, 2018). This is also something to consider.

Altogether, our results show that plastic material releases a variety of known and unknown substances, leading to a complex mixture of chemicals with potentially harmful effects. UV radiation can degrade plastic material and can increase release of substances from this material. Environmental impacts and possible human health concerns need further investigations. Not just new but also aged materials should be tested under different weathering conditions. It seems that metabolization of released substances play a role in their toxicological potential. Therefore, metabolization should be considered for risk assessment analyses.

##  Supplemental Information

10.7717/peerj.15192/supp-1 Supplemental Information 1 Supplementary Information.

10.7717/peerj.15192/supp-2 Supplemental Information 2 Raw Data ER Calux.

10.7717/peerj.15192/supp-3 Supplemental Information 3 Raw Data P53 Calux.

10.7717/peerj.15192/supp-4 Supplemental Information 4 Raw Data Umu test.

## Additional Information and Declarations

### Data Deposition

The following information was supplied regarding data availability: The data is available in the Supplementary Files.
