# Supplementary material for: Chemical and toxicological assessment of leachates from UV-degraded plastic materials using in-vitro bioassays
Source: PeerJ. 2023 Apr 11;11:e15192. doi: 10.7717/peerj.15192 (PMC10103695; doi:10.7717/peerj.15192)
Supplement: Supplemental Information 1 [file peerj-11-15192-s001.docx]

**Supplementary Information**

*Schwarz et al. (2022): Chemical and toxicological assessment of leachates and leached substances from UV-degraded plastic species using in-vitro bioassays*

**Table S1**: Number of plastic pellets (no.) and its surface-area-to-volume ratio (sa:vol) (mean ± SD) [cm^2^/L^-1^] (equivalent to 100 g/L^-1^) (taken from Klein et al., 2021)

| Sample | No. | Sa:vol [cm^2^ L^-1^] |
| --- | --- | --- |
| PP-H | 4500 | 2469 ± 291 |
| PP-C | 4716 | 3120 ± 545 |
| PET-A | 5686 | 1821 ± 123 |
| PET-R | 4870 | 1673 ± 165 |
| PS-GP | 5044 | 2075 ± 202 |
| PS-HI | 4154 | 1978 ± 206 |
| LDPE | 3689 | 2274 ± 119 |
| LDPE-R | 3014 | 2395 ± 442 |
| PVC-A | 1507 | 1273 ± 130 |
| PVC-R | 848 | 945 ± 100 |
| Bio-PBS | 6890 | 2074 ± 120 |
| SB | 4080 | 1619 ± 448 |

**Table S2**: Water parameters (pH and conductivity) after the leaching procedure. Prior to procedure, the leaching medium had an electrical conductivity of <0.055 µS/cm^-1^. T1 = dark control, T2 = UV-C, T3 = UV-A/B, T4 = UV-A/B_aq_, C = control (n = 3) (taken from Klein et al., 2021)

| Sample | T1 | | T2 | | T3 | | T4 | |
| --- | --- | --- | --- | --- | --- | --- | --- | --- |
|  | pH | Conductivity  [µS cm^-1^] | pH | Conductivity  [µS cm^-1^] | pH | Conductivity  [µS cm^-1^] | pH | Conductivity  [µS cm^-1^] |
| C | 5.45 | 8.41 | 6.03 | 11.7 | 6.18 | 9.30 | 6.03 | 16.9 |
| PP-H | 4.26 | 4.76 | 8.89 | 6.18 | 7.14 | 7.85 | 8.05 | 8.22 |
| PP-C | 5.76 | 4.04 | 4.97 | 5.06 | 6.90 | 5.91 | 5.31 | 8.37 |
| PET-A | 5.15 | 3.82 | 7.98 | 8.11 | 4.19 | 10.0 | 6.56 | 21.2 |
| PET-R | 5.12 | 3.48 | 7.95 | 9.29 | 4.21 | 8.61 | 6.26 | 9.51 |
| PS-GP | 5.20 | 4.45 | 4.59 | 8.48 | 6.66 | 8.75 | 7.87 | 8.27 |
| PS-HI | 5.75 | 4.50 | 4.53 | 9.69 | 7.78 | 7.30 | 4.23 | 18.5 |
| LDPE | 5.02 | 3.41 | 4.87 | 4.47 | 8.08 | 6.30 | 6.35 | 14.0 |
| LDPE-R | 7.13 | 5.93 | 6.68 | 6.76 | 6.86 | 7.36 | 6.76 | 9.51 |
| PVC-A | 5.43 | 9.95 | 6.75 | 12.3 | 7.24 | 8.68 | 5.12 | 15.1 |
| PVC-R | 5.67 | 17.2 | 7.82 | 10.2 | 7.65 | 18.5 | 5.67 | 24.2 |
| Bio-PBS | 4.70 | 7.06 | 8.06 | 38.1 | 4.00 | 12.8 | 5.58 | 21.9 |
| SB | 6.21 | 76.8 | 5.86 | 65.8 | 6.04 | 85.7 | 5.61 | 82.7 |

**Table S3**: Results of chemical analysis (counts and intensities) of investigated samples at different treatment methods (T1-T4)

| Sample | Weathering | | positive mode | | negative mode | |
| --- | --- | --- | --- | --- | --- | --- |
|  |  |  | counts | intensity | counts | intensity |
| PP-H | T1 | DC | 27 | 10501 | 19 | 7978 |
|  | T2 | UV-C | 16 | 6657 | 21 | 9385 |
|  | T3 | UV-A/B | 21 | 8863 | 43 | 17900 |
|  | T4 | UV-A/Baq | 43 | 17726 | 35 | 20103 |
| PP-C | T1 | DC | 41 | 22541 | 45 | 19907 |
|  | T2 | UV-C | 50 | 24187 | 52 | 19609 |
|  | T3 | UV-A/B | 45 | 31166 | 33 | 14411 |
|  | T4 | UV-A/Baq | 90 | 52970 | 68 | 37438 |
| PET-A | T1 | DC | 49 | 3684 | 53 | 14476 |
|  | T2 | UV-C | 288 | 34842 | 750 | 325296 |
|  | T3 | UV-A/B | 53 | 3189 | 62 | 14575 |
|  | T4 | UV-A/Baq | 47 | 4062 | 72 | 21395 |
| PET-R | T1 | DC | 28 | 1750 | 31 | 3949 |
|  | T2 | UV-C | 290 | 35636 | 713 | 327823 |
|  | T3 | UV-A/B | 51 | 5259 | 43 | 5298 |
|  | T4 | UV-A/Baq | 54 | 2446 | 49 | 8837 |
| PS-GP | T1 | DC | 17 | 839 | 26 | 1332 |
|  | T2 | UV-C | 355 | 30195 | 512 | 66268 |
|  | T3 | UV-A/B | 77 | 3586 | 41 | 1835 |
|  | T4 | UV-A/Baq | 41 | 3126 | 79 | 4961 |
| PS-HI | T1 | DC | 38 | 2366 | 42 | 4009 |
|  | T2 | UV-C | 359 | 28328 | 531 | 65026 |
|  | T3 | UV-A/B | 40 | 3125 | 72 | 5151 |
|  | T4 | UV-A/Baq | 51 | 2836 | 79 | 7123 |
| LDPE | T1 | DC | 106 | 4060 | 134 | 9318 |
|  | T2 | UV-C | 137 | 6115 | 143 | 12662 |
|  | T3 | UV-A/B | 91 | 3996 | 107 | 7505 |
|  | T4 | UV-A/Baq | 84 | 4704 | 78 | 6485 |
| LDPE-R | T1 | DC | 2390 | 723988 | 1085 | 278723 |
|  | T2 | UV-C | 2876 | 856116 | 1685 | 481117 |
|  | T3 | UV-A/B | 2665 | 768544 | 1244 | 305119 |
|  | T4 | UV-A/Baq | 3178 | 1107703 | 1436 | 370568 |
| PVC-A | T1 | DC | 696 | 64295 | 316 | 26636 |
|  | T2 | UV-C | 2910 | 610491 | 1603 | 539553 |
|  | T3 | UV-A/B | 2853 | 477515 | 1531 | 409501 |
|  | T4 | UV-A/Baq | 2771 | 405109 | 1374 | 266493 |
| PVC-R | T1 | DC | 938 | 97464 | 487 | 76842 |
|  | T2 | UV-C | 1305 | 132378 | 1122 | 147017 |
|  | T3 | UV-A/B | 1015 | 86083 | 741 | 114708 |
|  | T4 | UV-A/Baq | 1103 | 105532 | 702 | 77095 |
| Bio-PBS | T1 | DC | 1167 | 2750102 | 943 | 1726553 |
|  | T2 | UV-C | 2183 | 3072620 | 1835 | 3004026 |
|  | T3 | UV-A/B | 1501 | 3398643 | 1206 | 2160582 |
|  | T4 | UV-A/Baq | 1683 | 4881702 | 1478 | 4021899 |
| SB | T1 | DC | 2081 | 1824419 | 2085 | 1474944 |
|  | T2 | UV-C | 2018 | 1306867 | 1777 | 1111502 |
|  | T3 | UV-A/B | 2412 | 2061482 | 2335 | 1711027 |
|  | T4 | UV-A/Baq | 2509 | 2596363 | 2364 | 2245342 |

***Cytotoxicity (ERα-cells)***

a.)


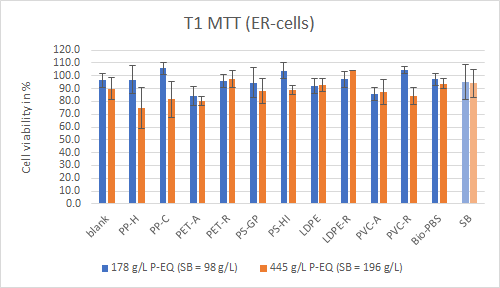


b.)


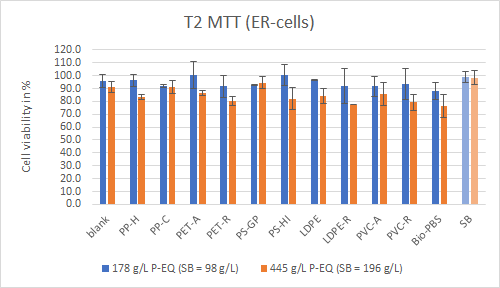


c.)


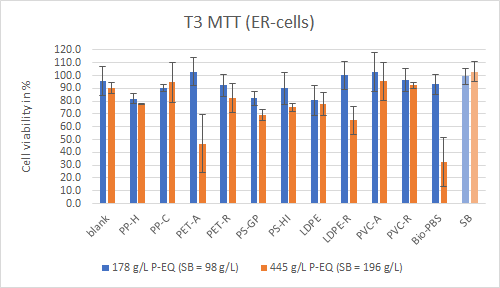


d.)


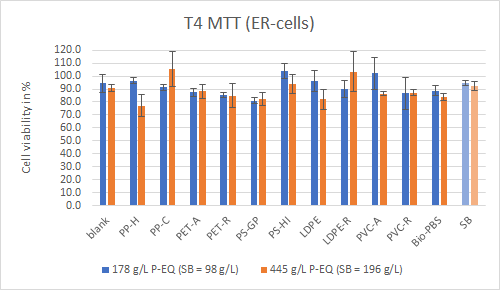


**Fig. S1**: Results of the MTT-Assay performed with ERα-cells at two different leachate concentrations. Cytotoxic effects were prominent at a cell viability < 70%.

***Cytotoxicity (p53-cells)***

a.)


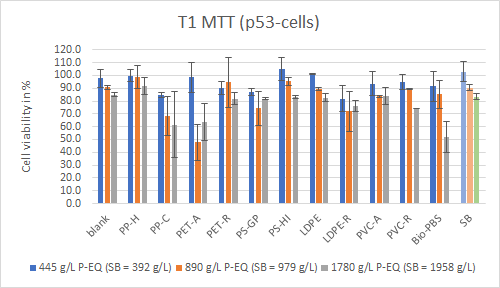


b.)


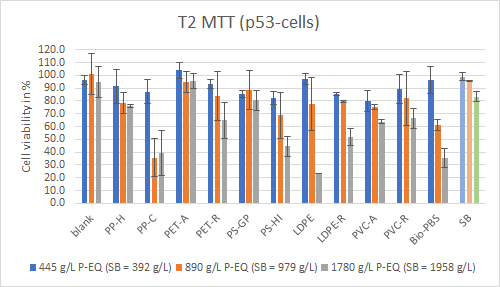


c.)


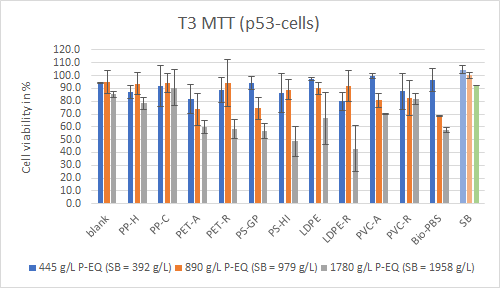


d.)


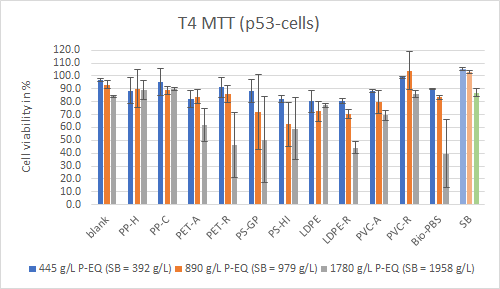


**Fig. S2**: Results of the MTT-Assay performed with p53-cells at three different leachate concentrations. Cytotoxic effects were prominent at a cell viability < 70%.

***Estrogenicity (ERα-cells)***

a.)


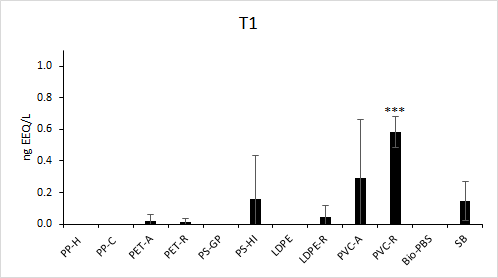


b.)


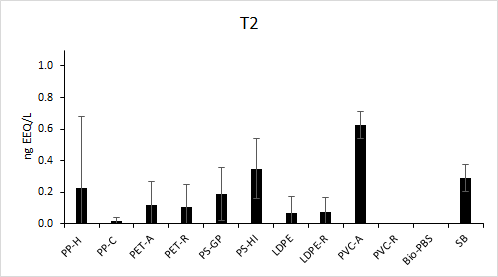


c.)


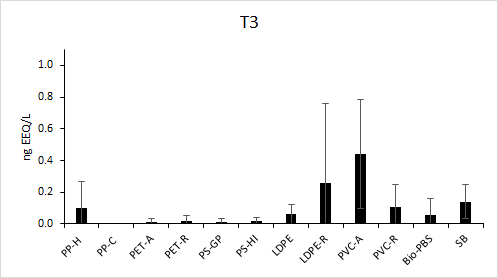


d.)


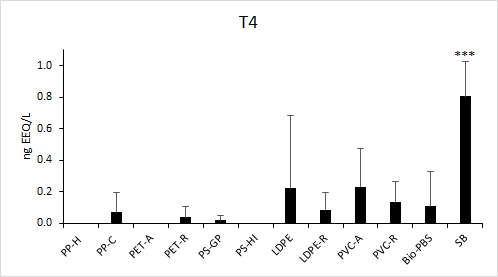


**Fig. S3**: Results of the estrogenicity measurements of plastic leachates at different treatment conditions (T1 – T4) using ER Calux. Exposure time: 24 h; Exposure concentrations: 178 g/L P-EQ (196 g/L P-EQ for SB). *** highly significant (p < 0.001)

***Genotoxicity (p53-Calux)***


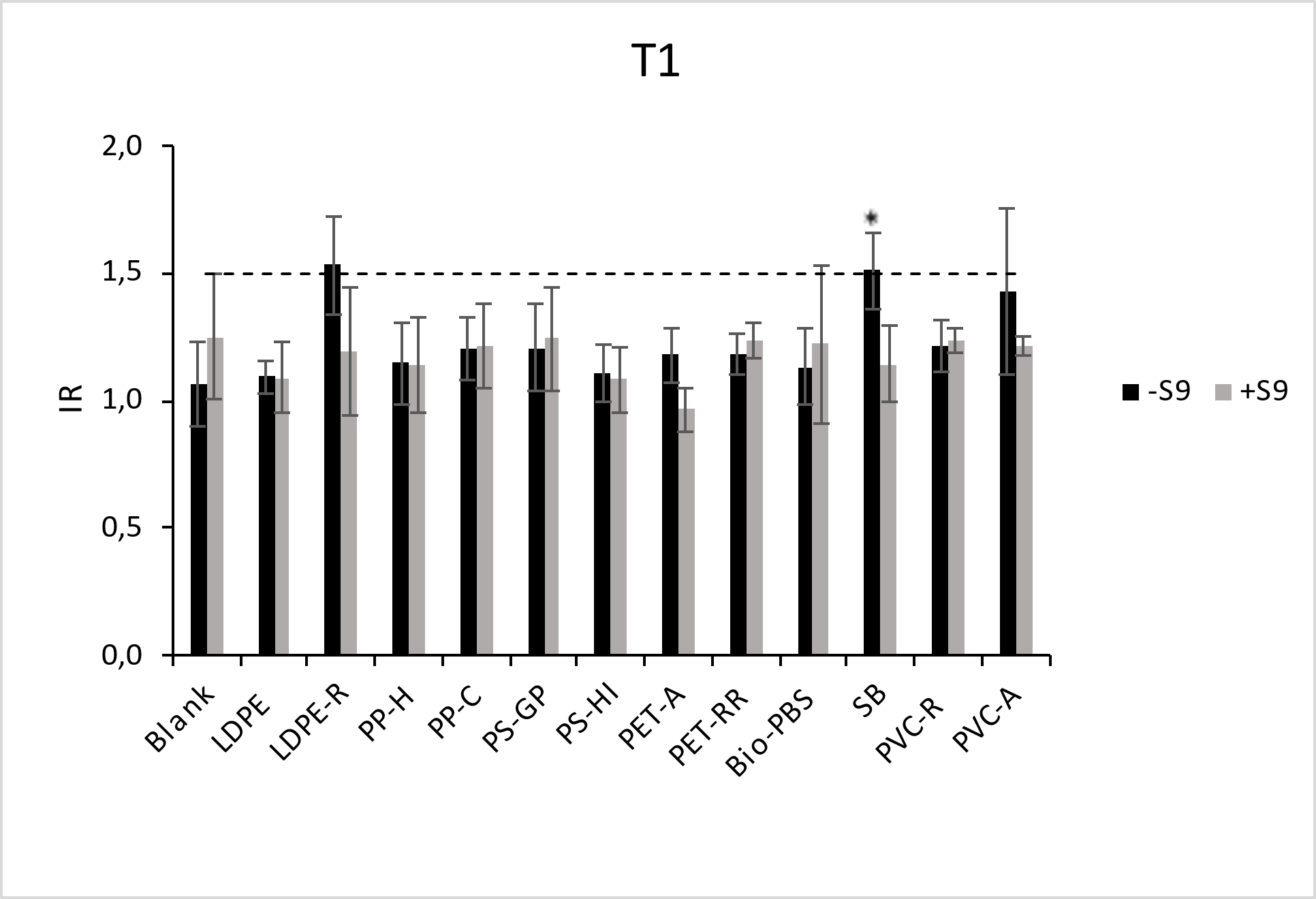


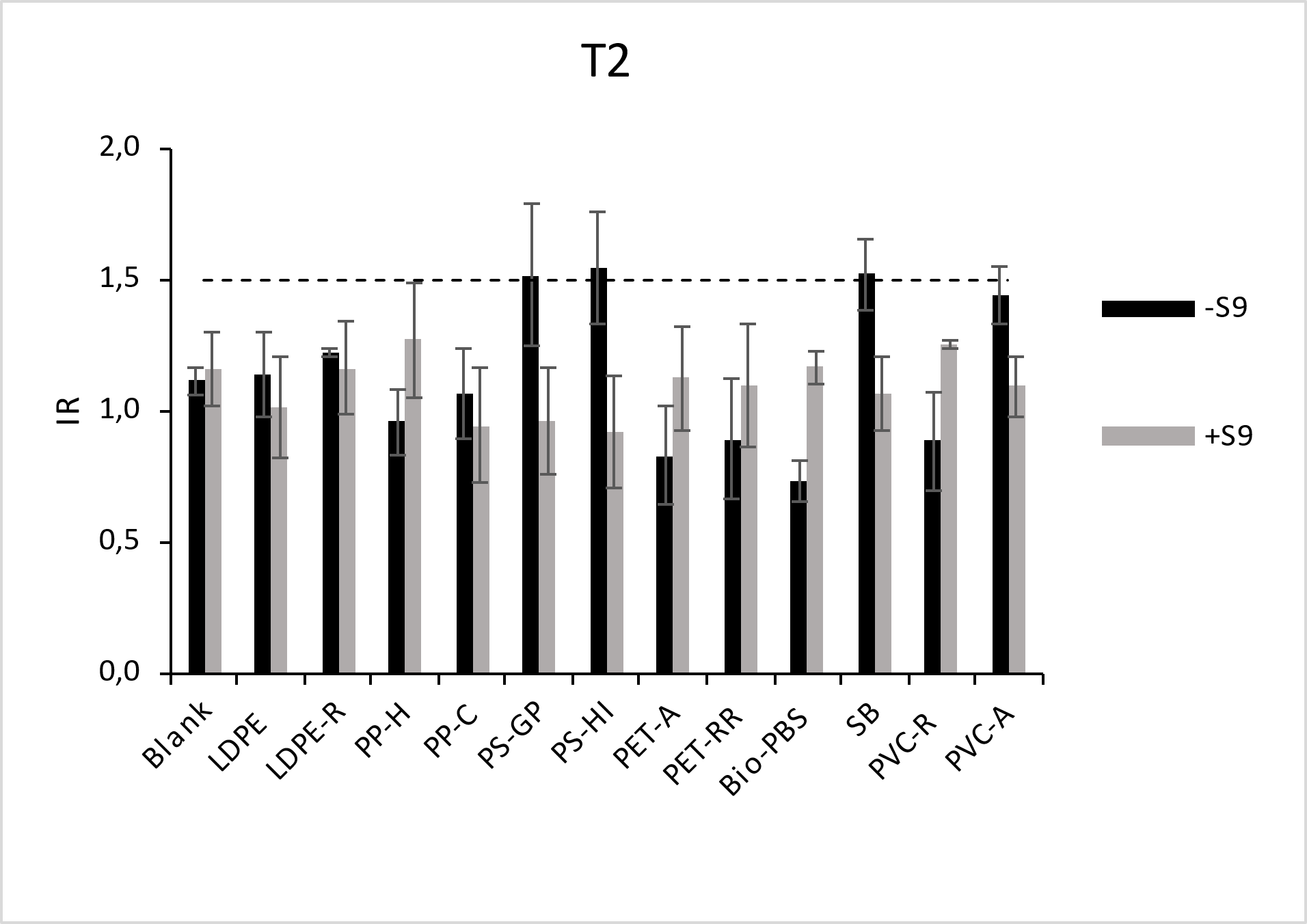


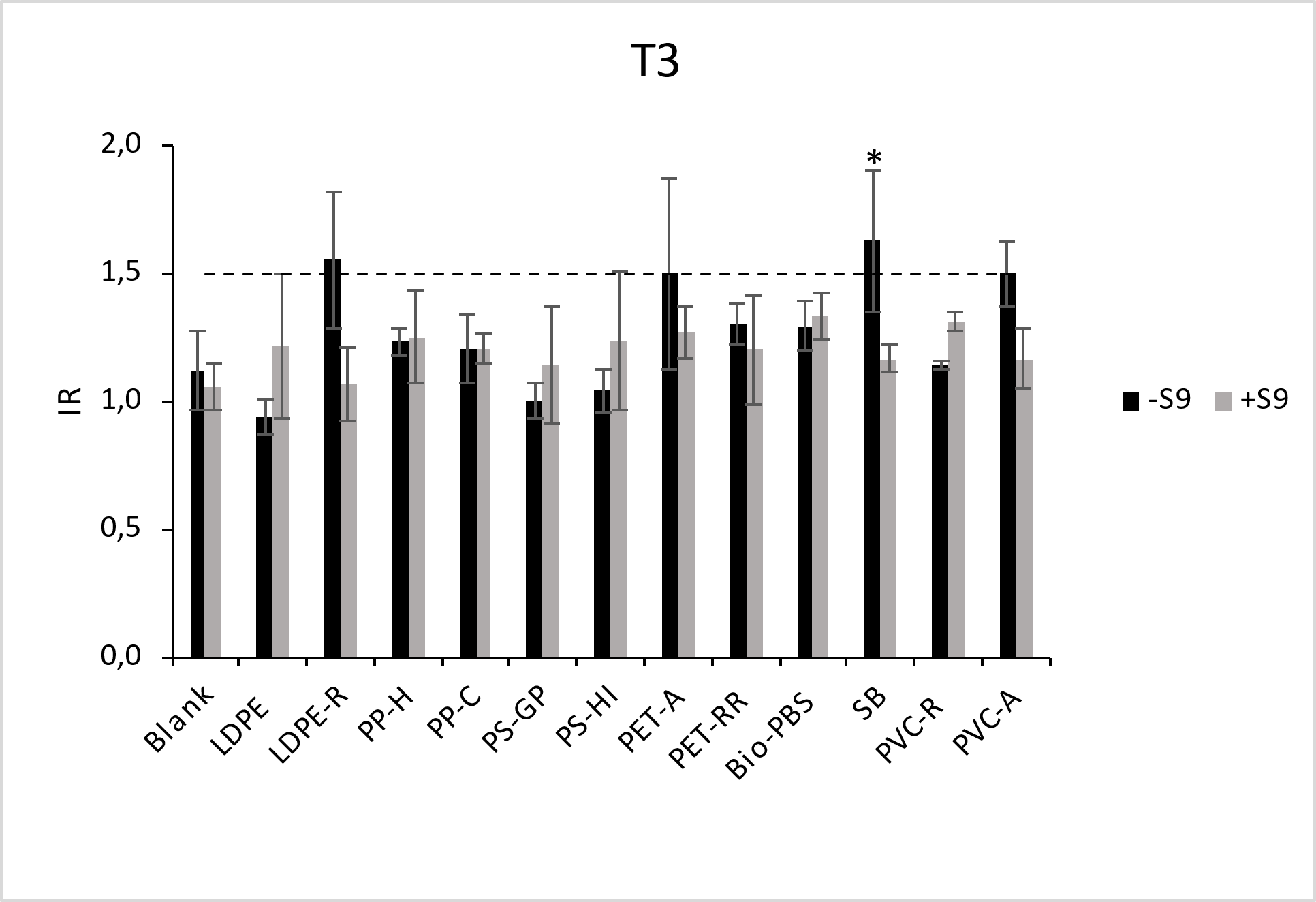


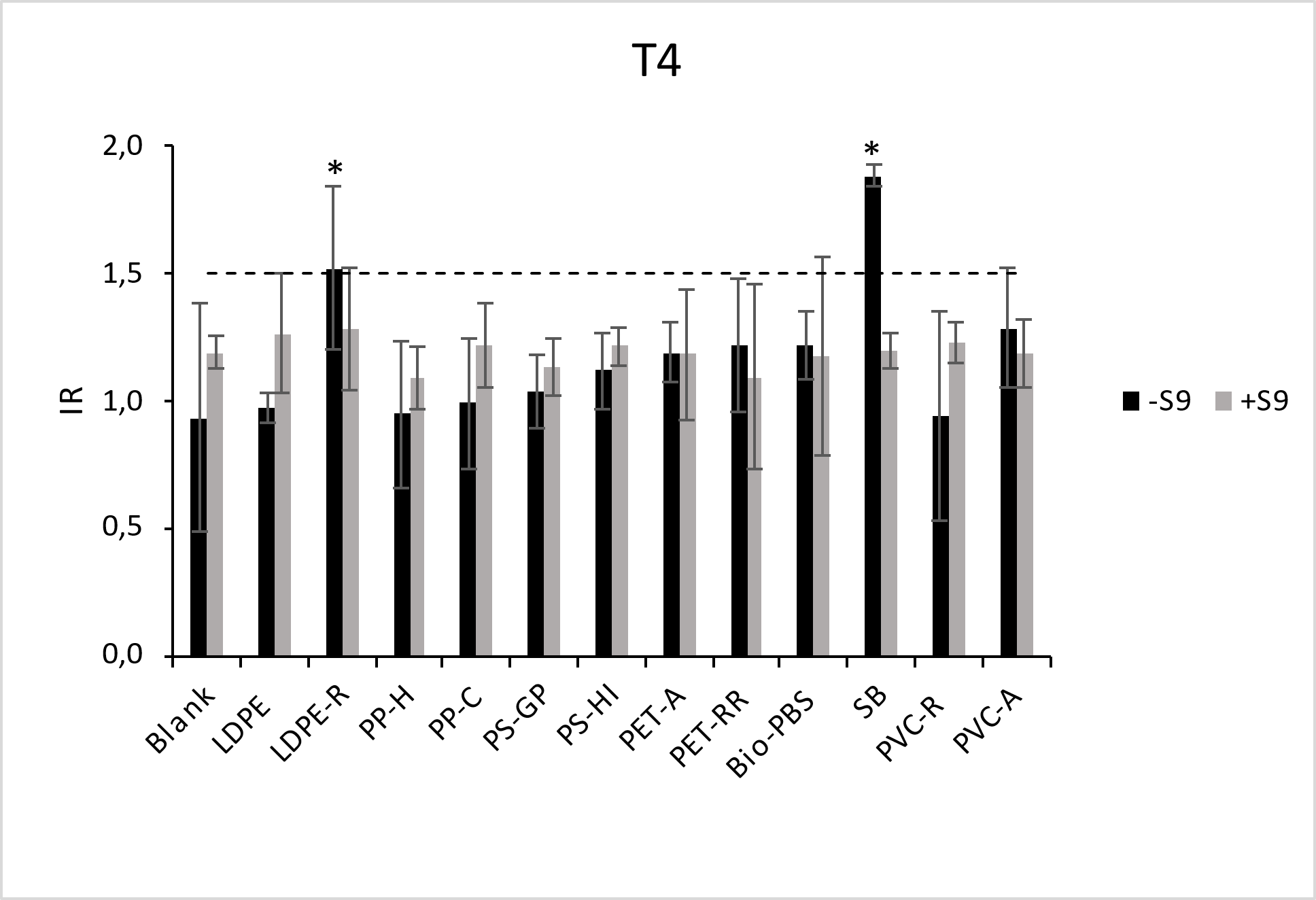


**Fig. S4**: Results of the p53-Calux (IR: Induction Rate; -S9: without S9-mix; +S9: with S9-mix)
